# Structure of the Intermediate Filament-Binding Region of Desmoplakin

**DOI:** 10.1371/journal.pone.0147641

**Published:** 2016-01-25

**Authors:** Hyunook Kang, Thomas M. Weiss, Injin Bang, William I. Weis, Hee-Jung Choi

**Affiliations:** 1 Dept. of Biological Sciences, Seoul National University, Seoul, South Korea; 2 SLAC National Laboratory, Menlo Park, California, United States of America; 3 Depts. of Structural Biology and Molecular & Cellular Physiology, Stanford University School of Medicine, Stanford, California, United States of America; Universitetet i Bergen, NORWAY

## Abstract

Desmoplakin (DP) is a cytoskeletal linker protein that connects the desmosomal cadherin/plakoglobin/plakophilin complex to intermediate filaments (IFs). The C-terminal region of DP (DPCT) mediates IF binding, and contains three plakin repeat domains (PRDs), termed PRD-A, PRD-B and PRD-C. Previous crystal structures of PRDs B and C revealed that each is formed by 4.5 copies of a plakin repeat (PR) and has a conserved positively charged groove on its surface. Although PRDs A and B are linked by just four amino acids, B and C are separated by a 154 residue flexible linker, which has hindered crystallographic analysis of the full DPCT. Here we present the crystal structure of a DPCT fragment spanning PRDs A and B, and elucidate the overall architecture of DPCT by small angle X-ray scattering (SAXS) analysis. The structure of PRD-A is similar to that of PRD-B, and the two domains are arranged in a quasi-linear arrangement, and separated by a 4 amino acid linker. Analysis of the B-C linker region using secondary structure prediction and the crystal structure of a homologous linker from the cytolinker periplakin suggests that the N-terminal ~100 amino acids of the linker form two PR-like motifs. SAXS analysis of DPCT indicates an elongated but non-linear shape with R_g_ = 51.5 Å and D_max_ = 178 Å. These data provide the first structural insights into an IF binding protein containing multiple PRDs and provide a foundation for studying the molecular basis of DP-IF interactions.

## Introduction

Desmosomes are intercellular junctions that confer structural integrity to tissues by linking the intermediate filament cytoskeletons of adjacent cells. Desmosomes contain the desmosomal cadherins (desmogleins and desmocollins), whose extracellular regions to form the adhesive bond between cells, and whose cytoplasmic regions link to intermediate filaments. The cytosolic armadillo proteins plakoglobin and plakophilins interact with desmosomal cadherins and with desmoplakin (DP), a member of the plakin family of cytolinkers [1, 2]. The N-terminal domain of plakophilin has been shown to interact with desmosomal cadherins and DP [3].

Human DP (Uniprot ID P15924) contains 2871 amino acids and contains three distinct regions. The N-terminal 1056-residue domain contains an N-terminal 175 residue domain of unknown structure, followed by six spectrin-like repeats [4–6]. This domain interacts with cadherins and desmosomal armadillo proteins. The central 903 residues (isoform 1) or 304 residues (isoform 2) form a coiled coil dimer. The 912 amino acid C-terminal region (DPCT) binds to intermediate filaments (IFs), including keratins, vimentin and desmin [7–10]. DPCT contains three plakin repeat domains (PRDs), designated PRD-A, PRD-B, and PRD-C, which share ~30% sequence identity with each other. The three DP PRDs each bind weakly to vimentin, and stronger binding is observed for the B-C and full ABC regions [10]. Other plakin family proteins, such as plectin, envoplakin, periplakin and bullous pemphigoid antigen-1, also interact with IFs but with different specificities and affinities [11–13]. These proteins, except for periplakin, contain at least one PRD: for example, plectin has five copies of PRD-B and one PRD-C, and envoplakin contains one PRD-C [14].

Few structural data are available for IF binding proteins, except for PRD-B and PRD-C of DPCT [10]. Crystal structures of PRD-B and PRD-C showed that they contain 4.5 copies of a plakin repeat (PR) and share structural similarity with RMSD value of 2.0 Å for 169 Cα positions. Canonical PRs (PRs 1 to 4) consist of a β-hairpin followed by two antiparallel α-helices, while PR5, which forms half a PR, lacks the last α-helix. Based on sequence similarity, PRD-A is expected to form a similar PRD structure.

In addition to the PRDs, a portion of the sequence that links the desmoplakin PRDs B and C is well conserved in the plakin family [11]. Indeed, periplakin lacks a full PRD but has region homologous to this linker at its C-terminus. Several studies have suggested that this linker is important for IF binding [11]. In particular, Nikolic et al. reported that about 50 amino acids in the linker region of plectin (amino acids 4262–4316) are essential for vimentin binding [12]. In another study, the fifth PRD-B and the following linker were shown to interact with desmin and vimentin, whereas the PRD or the linker separately did not associate with desmin [13].

Here, we analyze the organization of the multiple PRDs in DPCT using x-ray crystallography and small-angle X-ray scattering (SAXS). PRDs A and B form a compact, but bent, structure in solution. Based on homology to periplakin, the linker between PRDs B and C is likely to contain some folded structure, and appears to form a roughly linear connection to PRD-C to create an extended overall structure.

## Materials and Methods

### Construct design and protein purification

Two constructs of DPCT, PRD-AB and PRD-ABC were expressed in *E*. *coli* DH5α cells using the pPROEX-HTc vector (Life Technologies) and purified as previously described [10]. Briefly, each protein was overexpressed with a tobacco etch virus (TEV) protease-cleavable His_6_ tag at its N-terminus. Protein was purified by Ni^2+^ affinity chromatography (Ni-NTA agarose, Qiagen), and the His_6_ tag removed by overnight treatment with TEV protease (20:1 substrate:TEV w/w) at 4°C. PRD-AB and PRD-ABC were subsequently purified by anion exchange chromatography (MonoQ, GE Healthcare) using a 50–350 mM NaCl gradient in Q buffer (20 mM Tris-Cl pH 8.0, 0.5 mM EDTA, 2 mM DTT), followed by size exclusion chromatography on Superdex S200 (GF buffer: 25 mM Tris-Cl pH 8.0, 100 mM NaCl, 2 mM DTT). Purified PRD-AB and PRD-ABC were concentrated to 10 mg ml^-1^ and used for SAXS data collection. For crystallization of PRD-AB, protein was concentrated to 30 mg ml^-1^.

### Crystallization and Structure determination of PRD-AB

Crystals of PRD-AB were grown by hanging drop vapor diffusion at 22°C by mixing protein solution with mother liquor of 22% mono-methyl polyethylene glycol 5000 (PEG MME 5K), 0.1 M MES (pH 6.5) and 0.2 M magnesium acetate. The crystals were flash frozen into liquid nitrogen using perfluoropolyether oil (PFO) as cryoprotectant. Diffraction data were measured at 100 K on beamline 11–1 at Stanford Synchrotron Radiation laboratory (SSRL), and processed with Mosflm and Scala [15, 16]. The crystals belong to space group P2_1_2_1_2_1_, and there is one molecule in the asymmetric unit. Data collection statistics are shown in Table 1.

**Table 1:** Crystallographic statistics for PRD-AB.

***Data collection***	
Wavelength (Å)	0.9537
Space group	P2_1_2_1_2_1_
Unit cell parameters *a*, *b*, *c* (Å)	111.93, 64.47, 74.04
Resolution (Å) (last shell)	50–2.6 (2.7–2.6)
Unique reflections	16648 (776)
Completeness (%)	97.5 (93.0)
Multiplicity	3.0 (2.9)
I/ (I)	10.6 (3.1)
R_merge_^a^	0.068 (0.35)
***Refinement***	
No. of reflections working set (test set)	15327 (1319)
R_cryst_/ R_free_^b^	0.21 / 0.26
bond length *rmsd* from ideal (Å)	0.002
bond angle *rmsd* from ideal (°)	0.50
***Ramachandran analysis***^c^	
% favored regions	95.5
% allowed regions	4.5
% outliers	0.0

*rmsd*, root-mean square deviation.

^a^R_merge_ = Σ_*h*_Σ_*I*_|I_*i*_*h*<I*h*>|Σ_*h*_Σ_*i*_(*h*), where I_*i*_(*h*) is the *i*^th^ measurement of reflection *h*, and <I(*h*)> is the weighted mean of all measurements of *h*.

^b^R = Σ_*h*_|F_obs_(*h*)| − |F_calc_(*h*)| | / Σ_*h*_|F_obs_(*h*)|. R_cryst_ and R_free_ were calculated using the working and test reflection sets, respectively.

^c^As defined in MolProbity

The structure of PRD-AB was solved by molecular replacement with Phaser [17], using the structure of PRD-B as a search model (PDB ID = 1LM7) [10]. The molecular replacement solution contained two copies of the PRD-B search model, which correspond to PRD-A and PRD-B. Iterative cycles of manual rebuilding with Coot [18] and refinement with Phenix [19] were performed to produce the final model, consisting of DPCT residues 1960–2448. The final refined model was validated by MolProbity (Table 1) [20].

### SAXS data analysis

SAXS experiments were performed at SSRL beamline 4–2 equipped with a Rayonix MX225HE CCD detector. Samples were measured at concentrations of 1, 2.5, 5, and 10 mg ml^-1^ in GF buffer containing 2% glycerol. SAXS data were collected at 15°C using an X-ray wavelength of 1.127 Å at a detector distance of 1.7 m, giving a measured range of 0.007 < q < 0.5 Å^-1^ (q = 4πsinθ/λ, where θ is the scattering angle and λ is the wavelength). Background scattering was subtracted and data were analyzed using ATSAS software package [21]. The radius of gyration (R_g_) for each protein was calculated by Guinier plot using the program PRIMUS [22] and the pair distribution function P(r) and the maximum particle size D_max_ were obtained by the program GNOM [23]. To generate *ab initio* envelopes, ten cycles of GASBOR [24] were run, followed by the program DAMAVER [25] to average the envelopes. The program CORAL [21] was used to improve the fit of the PRD-AB model to the experimental data. The input model was the crystallographic model of PRD-AB with the 4 amino acid linker between PRDs A and B replaced by four dummy residues. No contact restraints between the two domains were imposed during modeling. The fit of the PRD-AB crystal structure and the CORAL-refined model to the experimental data was evaluated by the χ value calculated from the program CRYSOL [26].

## Results and Discussion

### Crystal structure of PRD-AB

Most plakin family proteins, except for periplakin, contain at least one PRD. DPCT consists of three PRDs, PRD-A, PRD-B, and PRD-C, of which crystal structures of PRD-B and PRD-C were previously determined [10]. Although PRD-A is expected to form a similar PRD structure based on sequence similarity, no direct structural data have been available. We were unable to obtain diffraction quality crystals of purified PRD-A, so a construct spanning PRDs-A and B (PRD-AB) was designed and expressed in *E*. *coli*.

**Fig 1:**
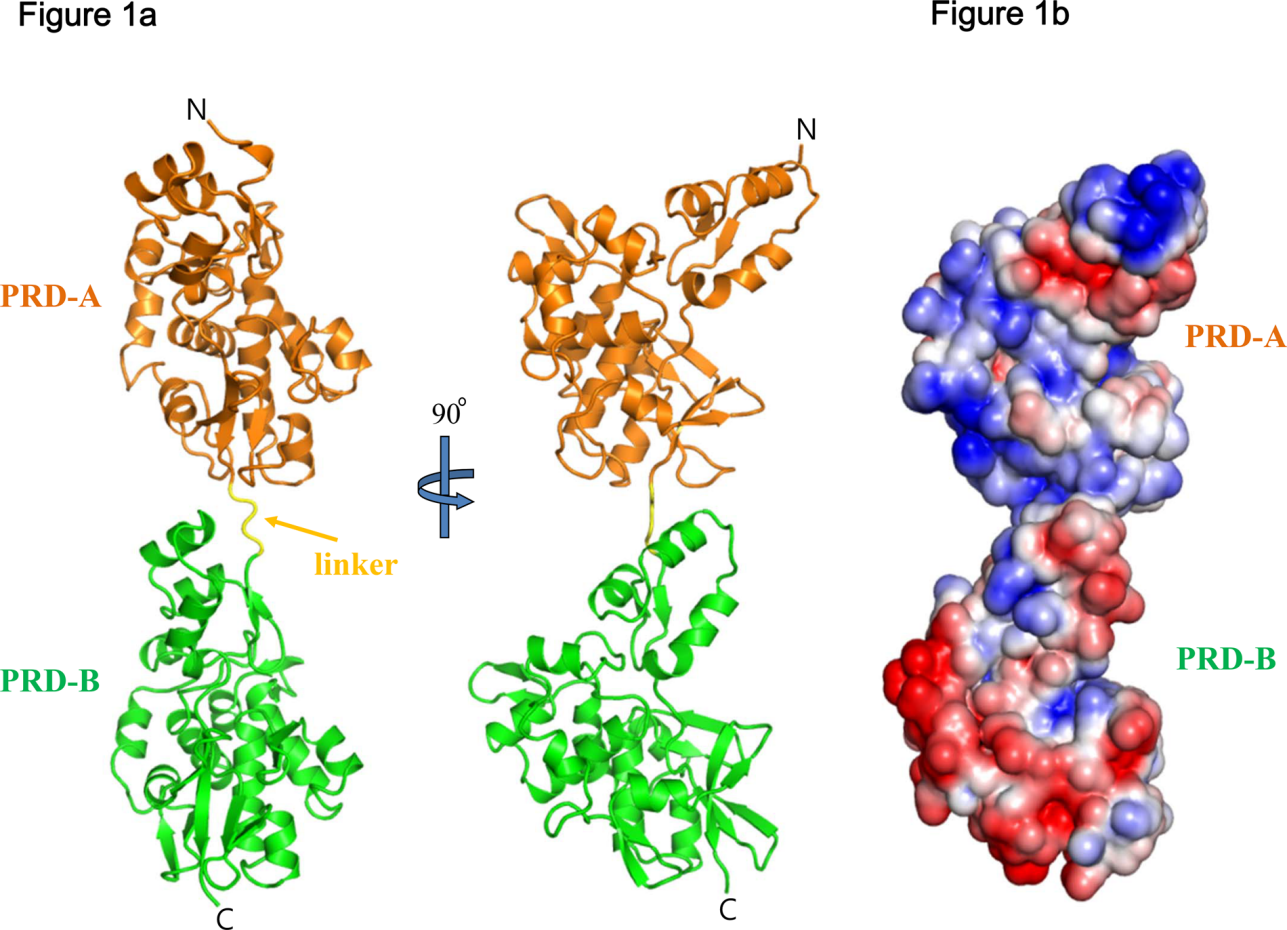
Overall structure of PRD-AB. (A) Domains A and B are colored orange and green, respectively. A 4 amino acid linker between domains A and B is shown in yellow. The structure of PRD-AB consisting of DPCT residues 1960–2448 is represented in two different views. (B) Electrostatic surface of the AB domain calculated with Pymol [27], with negative and positive regions colored red and blue, respectively. Contoured at ± 5 k_B_T/*e*.

We determined the crystal structure of PRD-AB consisting of residues 1960–2248 at 2.6 Å resolution (Table 1; Fig 1A). There is one copy in the asymmetric unit, consistent with size exclusion chromatography indicating that it is a monomer in solution. The two domains are arranged in a “beads on a string” manner to form an elongated structure with approximate overall molecular dimensions 38 Å × 42 Å × 108 Å (Fig 1A). Domain B is related to domain A by a 54 Å translation along a common long axis of the two domains, and a rotation of 29°. The two domains are connected by a 4 amino acid linker (residues 2204–2207) but do not form any direct contacts (interatomic distance < 4 Å) with one another (S1 Fig). Although this short linker is visible in the structure, it may have flexibility in solution. In fact, the amino acids comprising the linker have higher temperature factors than nearby residues and do not form direct contacts with either domain. However, since this linker is short, its flexibility is likely limited so as to avoid steric clashes between domains A and B. The flexibility of the linker between domains A and B is discussed further below.

PRD-A is found in desmoplakin but no other plakin family members. Although the overall structure of PRD-A is similar to that of PRD-B, it has a markedly different surface charge profile. Whereas domains B and C are acidic proteins (calculated pIs ~ 4.7 and 5.8), domain A is highly basic (pI ~9.0). As in PRD-B and PRD-C, a positively charged groove is also present in PRD-A; this groove was speculated to form a binding site for IFs [10]. The positively charged grooves present in domains A and B are separated by 56 Å, and there are two separate basic and acidic charged regions from domains A and B, respectively (Fig 1B). It is not clear whether this charge distribution is important for desmoplakin function in vivo. Previous studies using co-sedimentation assays with vimentin demonstrated that PRD-A binds to vimentin weakly, but comparably, to the other individual PRDs. Interestingly, PRD-AB doesn’t bind to vimentin as strongly as constructs containing the conserved linker between PRD-B and PRD-C, *i*.*e*., PRD-B+linker, PRD-B through PRD-C, or the entire PRD region containing PRDs A, B, and C [10]. These data may indicate the need for a long linker to enable simultaneous binding of multiple PRDs to an IF.

**Fig 2:**
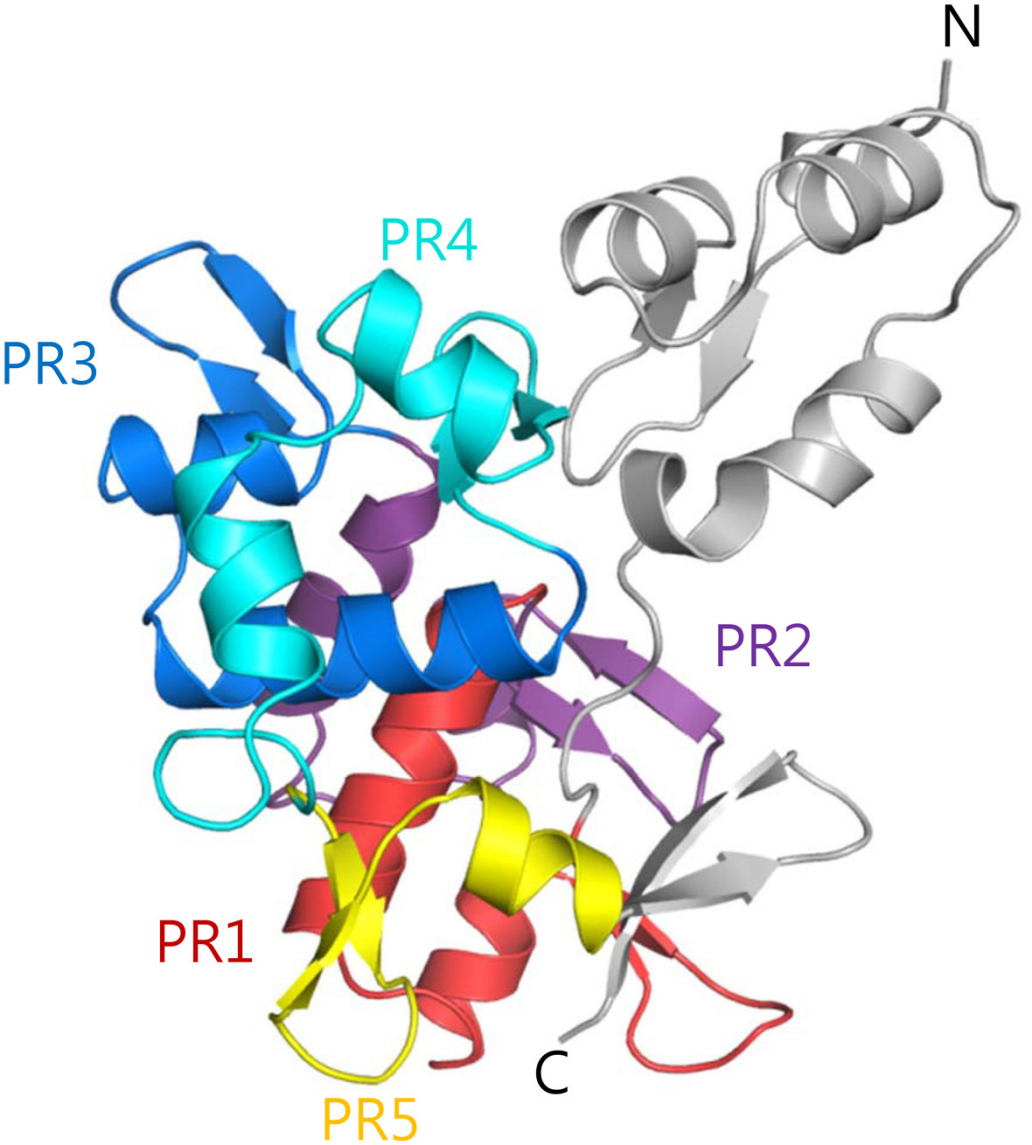
Overall structure of PRD-A. Each plakin repeat is colored differently and labeled as PR1 to PR5. The N-terminal PR like motif and the C-terminal region are colored grey.

As expected from sequence analysis, the structure of PRD-A is very similar to that of PRD-B (Fig 2). It consists of 4.5 PRs and an extra N-terminal PR like motif. Superposition of the three domains of DPCT shows that their overall structures are very similar. The principal difference among PRDs A, B and C is the presence of an additional N-terminal PR-like motif in PRD-A and –B that protrudes from the globular domain formed by the 4.5 PRs (grey in Fig 2). This motif features a β-hairpin followed by three antiparallel α-helices, but the second α-helix is much shorter than in canonical PR and is followed by another α-helix. The N-terminal PR like motifs of domains A and B align very well, with an RMSD value of 1.0 Å for 49 Cα atoms. Although the three dimensional structures of other plakin proteins are not available, amino acid sequence analysis suggest that the N-terminal PR like motif is present in PRD-B domains found in other plakin family members (PRD-A is unique to DP).

### Structural analysis of the B-C linker

The crystal structures of the desmoplakin PRDs represent 78% of the DPCT sequence, but the structure of the 154 amino acid region that links PRDs B and C (designated here “B-C linker”; residues 2457–2608) is not known, making it difficult to assess how the three IF-binding PRDs are spatially organized in the full length protein and how the linker might contribute to IF binding. Attempts to crystallize purified PRD-BC and PRD-ABC failed, which is likely due to flexibility in the linker, as suggested by its proteolytic sensitivity [10].

**Fig 3:**
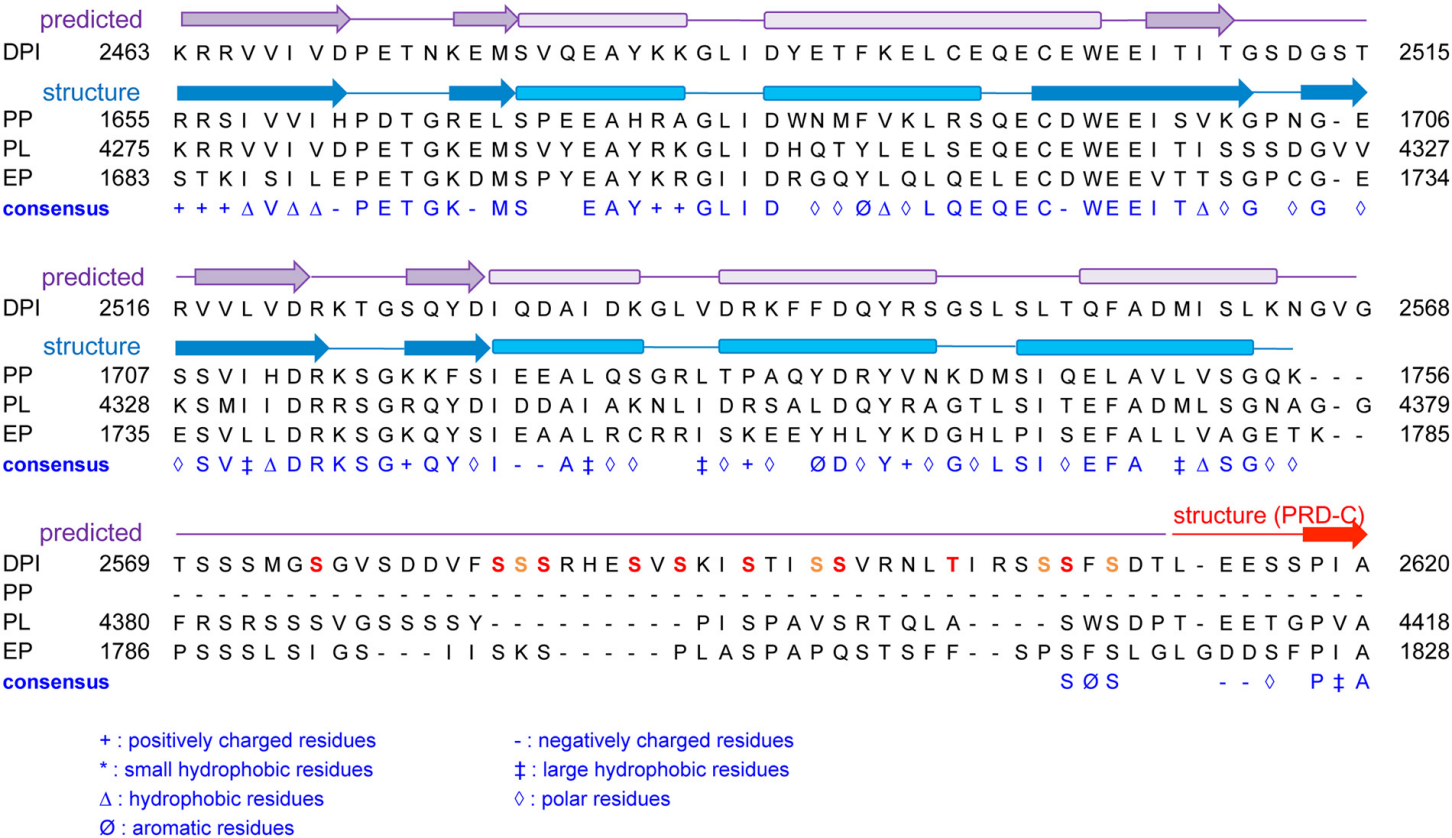
Linker region sequences. Sequence alignment of human desmoplakin I (DPI), human plectin (PL), human envoplakin (EP), and human periplakin (PP) in the region corresponding to the linker between PRDs B and C of desmoplakin. Secondary structures predicted with JPred4 are shown as arrows (β-strands) and thickened rectangles (α-helices). Ser/Thr residues in red and orange are predicted potential phosphorylation sites with scores higher than 0.9 and 0.6, respectively, by the NetPhos 2.0 server. The starting and ending residue numbers are indicated. A consensus sequence is shown at the bottom of each alignment. A consensus residue or class of residues, represented as a symbol, is indicated when more than 3/4 of the residues fall into this category.

Secondary structure prediction indicates that the N-terminal ~100 amino acids of the B-C linker are structured, whereas the C-terminal serine-rich sequence is not (Fig 3) [28]. This structured region is conserved among desmoplakin, plectin, and envoplakin with ~70% similarity (Fig 3). Interestingly, periplakin, which lacks a full PRD, has the homologous ~100 amino acids structured region at its C-terminus.

**Fig 4:**
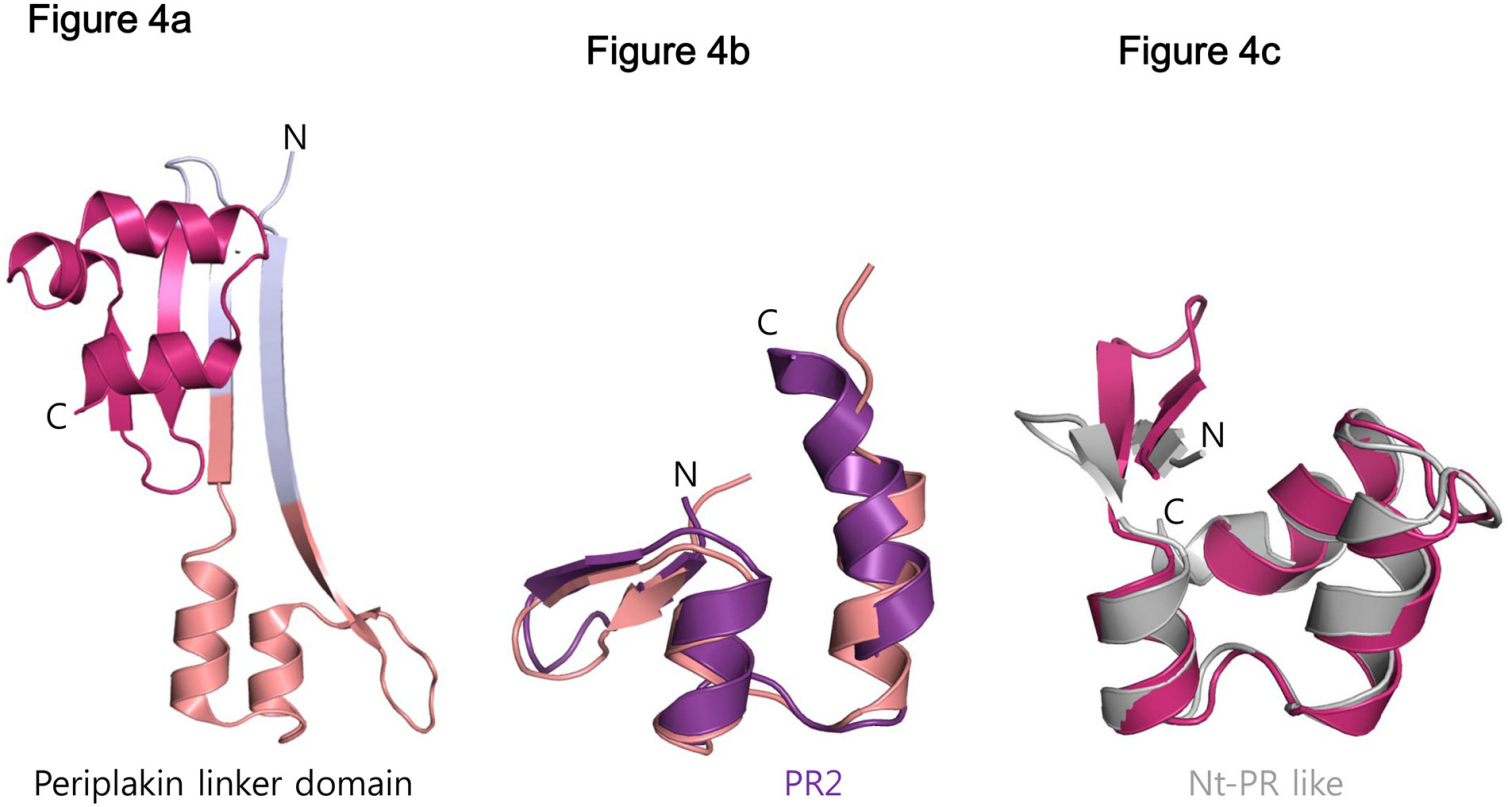
Structural analysis of the PRD-B-C linker region. (A) Crystal structure of the periplakin linker domain (PDB ID 4Q28). One protomer out of four molecules in the asymmetric unit is shown. (B) Structural alignment of the N-terminal part of periplakin linker domain with PR2 of PRD-A. (C) Structural alignment of the C-terminal part of periplakin linker domain with the N-terminal PR like motif of PRD-A.

The crystal structure of a portion of the corresponding region of periplakin has been deposited in the Protein Data Bank (ID 4Q28), and is consistent with the predicted secondary structure of the linker (Figs 3 and 4A). This fragment contains two PR-like motifs (Fig 4). The periplakin N-terminal PR-like motif structure aligns well with a canonical PR2 repeat, except that the second α helix is shorter in periplakin (Fig 4B). Curiously, secondary structure prediction of the desmoplakin B-C linker suggests that this α helix is five residues longer, which would extend into the region that forms a second β-strand in periplakin (Fig 3). In the periplakin structure, an N-terminal polyhistidine affinity tag forms an extended β-strand that pairs with this β-strand (Fig 4B). It is not clear whether this central β-strand is favored by pairing with the N-terminal polyhistidine tag.

The C-terminal PR-like motif of the periplakin linker aligns well with the N-terminal PR-like motif found in the desmplakin PRDs A and B, except for the different relative position of the β hairpin (Fig 4C). Because the two strands of this β hairpin contribute to the central β sheet formed with the N-terminal His_6_ tag, their position may be influenced by the presence of the affinity tag. The secondary structure prediction of the desmoplakin B-C linker indicates that the two PR-like motifs would be separated by a 7 amino acid loop (residues 2510–2516; Fig 3), whereas the two motifs are connected by two amino acids in the periplakin structure. Therefore, it seems likely that DPCT linker forms two separate structural modules, one PR motif and one PR-like motif (*i*.*e*., the N-terminal PR-like motif of PRD-A or PRD-B), separated by an unstructured loop. Previous studies of plectin showed that the linker is important for IF binding, but used several different ranges of the linker region without considering the structural motifs in the linker region [12, 13]. Based on the conservation and predicted structure, we suggest that IF binding activity in the linker maps to the structured, PR-like motifs.

### Overall structure of DPCT

**Fig 5:**
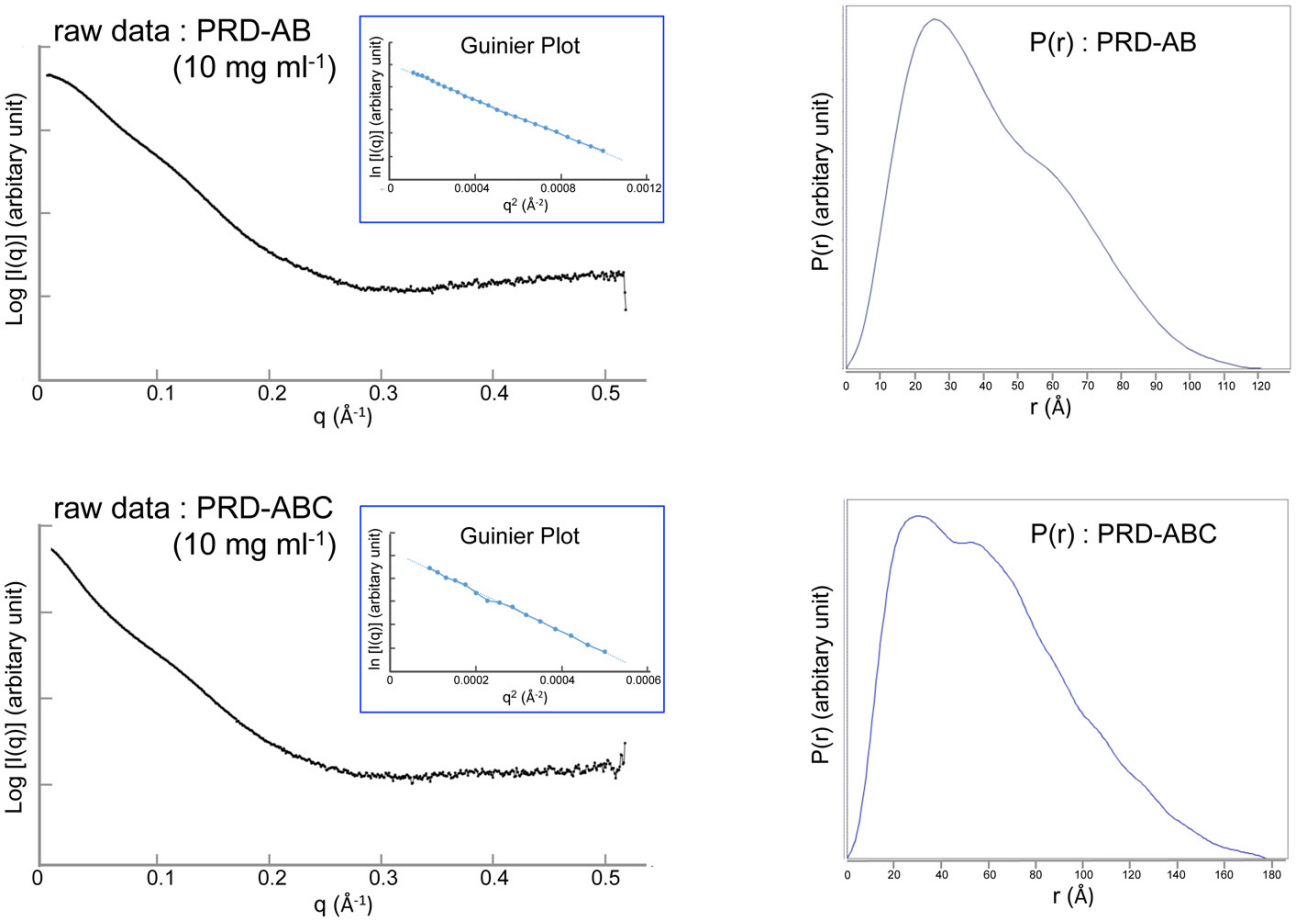
SAXS data and analyses of PRD-AB and PRD-ABC. Raw data at the highest concentration with an inset of the Guinier plot and P(r) of PRD-AB and PRD-ABC are shown.

**Fig 6:**
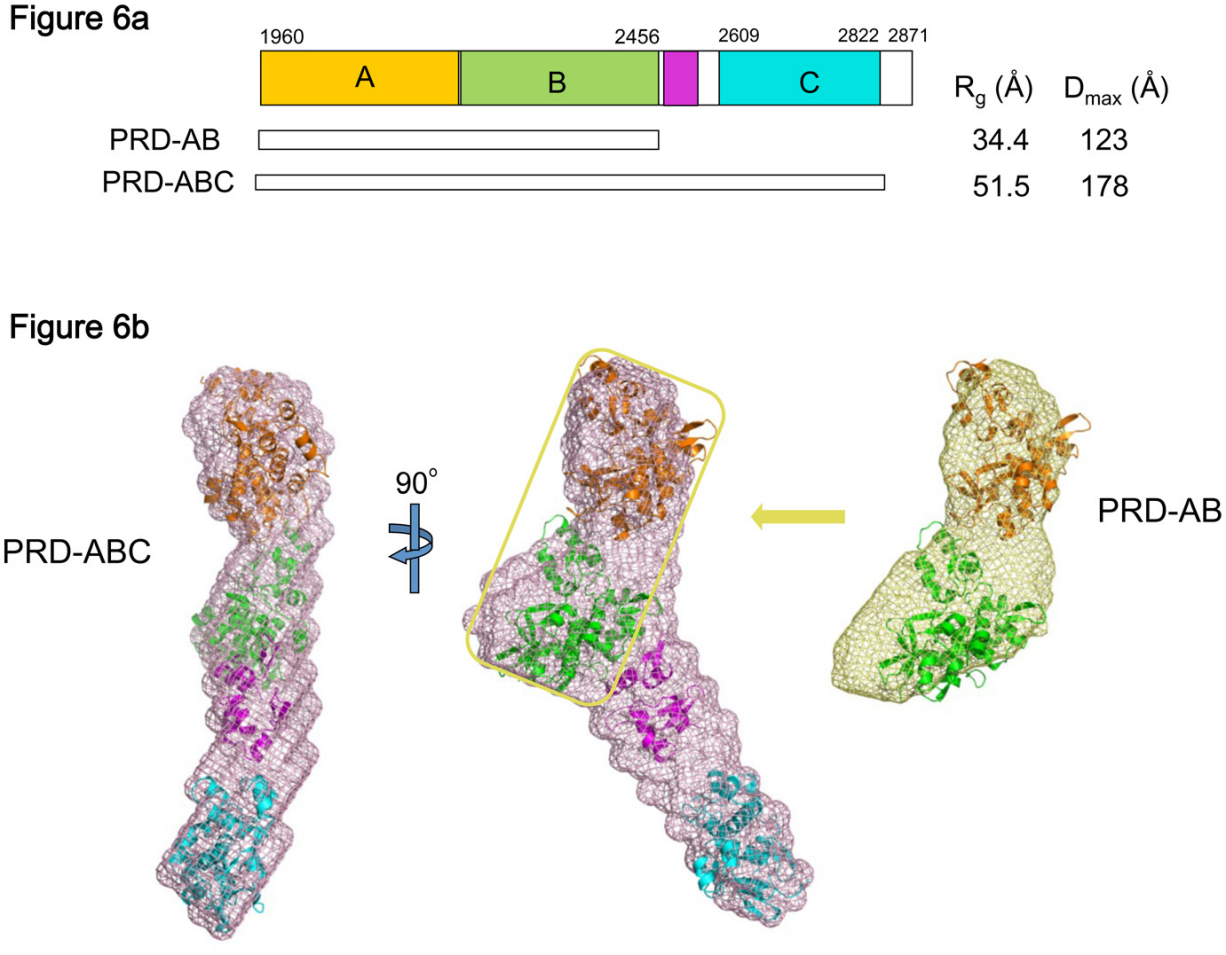
SAXS analysis of DPCT. (A) Construct information and the values of R_g_ and D_max_ from SAXS data analyses. (B) Solvated molecular envelopes of PRD-AB and PRD-ABC. The PRD-AB CORAL model and the crystal structures of periplakin linker and PRD-C are modeled into PRD-ABC envelope.

To visualize the overall architecture of DPCT spanning the three IF-binding PRDs, small angle X-ray scattering (SAXS) experiments were performed (Table 2). To assist in positioning three domains A, B, and C within the molecular envelope of PRD-ABC, SAXS data were measured from both the PRD-ABC and PRD-AB fragments. The quality of scattering data of proteins was good in the protein concentration range of 1 mg ml^-1^ to 10 mg ml^-1^ (Fig 5). The calculated radii of gyration (R_g_) were independent of protein concentration, and the Guinier plot showed a linear fit, suggesting that there is no inter-particle interaction (S2 Fig). In each Guinier plot, a straight line was obtained only for values q*R_g_, much less than 1.3, indicating that the scattering particles are elongated rather than close to spherical [29–31]. The Kratky plot, exhibiting a clear maximum at low q with a slightly elevated plateau at high q, indicates that the fragment is well folded and has only limited flexibility in solution (S2 Fig)[32]. The values of R_g_ and the maximum particle size (D_max_) are shown in Fig 6A. The overall shape of PRD-AB in solution, which was generated using the GASBOR [24] and DAMAVER package [25] was compared with the crystal structure. The envelope clearly showed that there are two domains but, in contrast to linear arrangement of domains A and B in the crystal structure, the SAXS envelope revealed a bent conformation (Fig 6B). Rigid body modeling with the program CORAL [21] was run to build a model of PRD-AB using the separate crystal structures of domains A and B. The four amino acid linker in the structure was replaced by a four-residue dummy atom linker, but no contact restraints between the two domains were imposed. The model generated by CORAL, although it still shows discrepancy from experimental data, improved the χ value from 13 to 3, calculated by the program CRYSOL [26]. In the CORAL model, PRD-A and PRD-B are closer together than in the crystal structure, with a calculated R_g_ of 34.4 Å, close to the experimental value and smaller than that of PRD-AB crystal structure (calculated R_g_ of 36 Å) (S3 Fig).

**Table 2:** SAXS data collection and derived parameters.

	PRD-AB	PRD-ABC
Data collection*		
instrument	SSRL BL4-2	
Defining slit size (H mm x V mm)	0.3 x 0.3	
Detector Distance (m)	1.7	
Wavelength (Å), energy (keV)	1.127, 11.0	
q-range (Å ^-1^)	0.007 ~ 0.5	
Exposure time	10 x 1s	
Temperature (K)	288.15	
Concentration (mg/ml)	1,2.5,5,10	
***Structural parameters***		
I(0) (cm^-1^, at 2.5mg/ml) from Guinier	0.11	0.16
R_g_(Å) from Guinier	34.0	50.8
R_g_(Å) from P(r)	34.4	51.5
D_max_(Å) from P(r)	123	178
R_g_(Å) from crystal structure	36.0	N/A
D_max_ (Å) from crystal structure	120	N/A
MW (kDa) (from sequence)	55	95
MW (kDa) (from Guinier)**	63	86
MW (kDa) (from Porod volume) ***	48	93
Software employed*		
Primary data collection and reduction	Blue-ICE, SASTOOL	
Data processing	PRIMUS	
Inverse Fourier Transform	GNOM	
*ab-initio* modelling	GASBOR, DAMAVER	
Rigid-body modeling	CORAL	
Computation of model intensities	CRYSOL	
3D graphics representation	PYMOL	

* These parameters where the same for all samples

** The deviation of molecular weights calculated from Guinier from the theoretical value was likely due to inaccurate estimates of protein concentration, which were determined by the absorbance at 280nm, possibly due to the presence of oxidized DTT in the sample buffer and small amounts of degradation products.

*** The Porod volume was calculated with DATPOROD v0.1a from the ATSAS release 2.7.

SAXS analysis of PRD-ABC resulted in an R_g_ value of 51.5 Å and a D_max_ of 178 Å. The molecular envelope of PRD-ABC was calculated and compared with those of PRD-AB, to locate each PRD domain. A structural model for PRD-ABC was built using the same PRD-AB model best fit to the PRD-AB SAXS data, the crystal structure of PRD-C and the linker structure (Fig 6B). As discussed above, it is likely that the B-C linker contains two PR-like motifs connected by an unstructured linker. Although the inability to crystallize the full PRD-ABC construct might imply some flexibility in the linker, it appears to be limited as shown by Kratky analysis (S2 Fig)[32]. The two periplakin linker motifs were separately located between PRD-B and PRD-C and the N-terminal and the C-terminal ends of each domain are reasonably positioned in this model. A 52 amino acid stretch that follows the structured linker region (residues 2564–2615) is missing in this model and it presumably occupies the unmodeled space between the structured part of the linker and PRD-C within this envelope. Attempts to model this region with CORAL produced models with these residues in a highly extended conformation that lie outside of the GASBOR-derived envelope. Given this inconsistency we cannot draw conclusions about its structure or degree of flexibility. Nonetheless, the models, as well as the model-independent SAXS parameters, suggest that DPCT adopts a fairly extended, nonlinear conformation.

### Positions of disease-associated DP mutations

**Fig 7:**
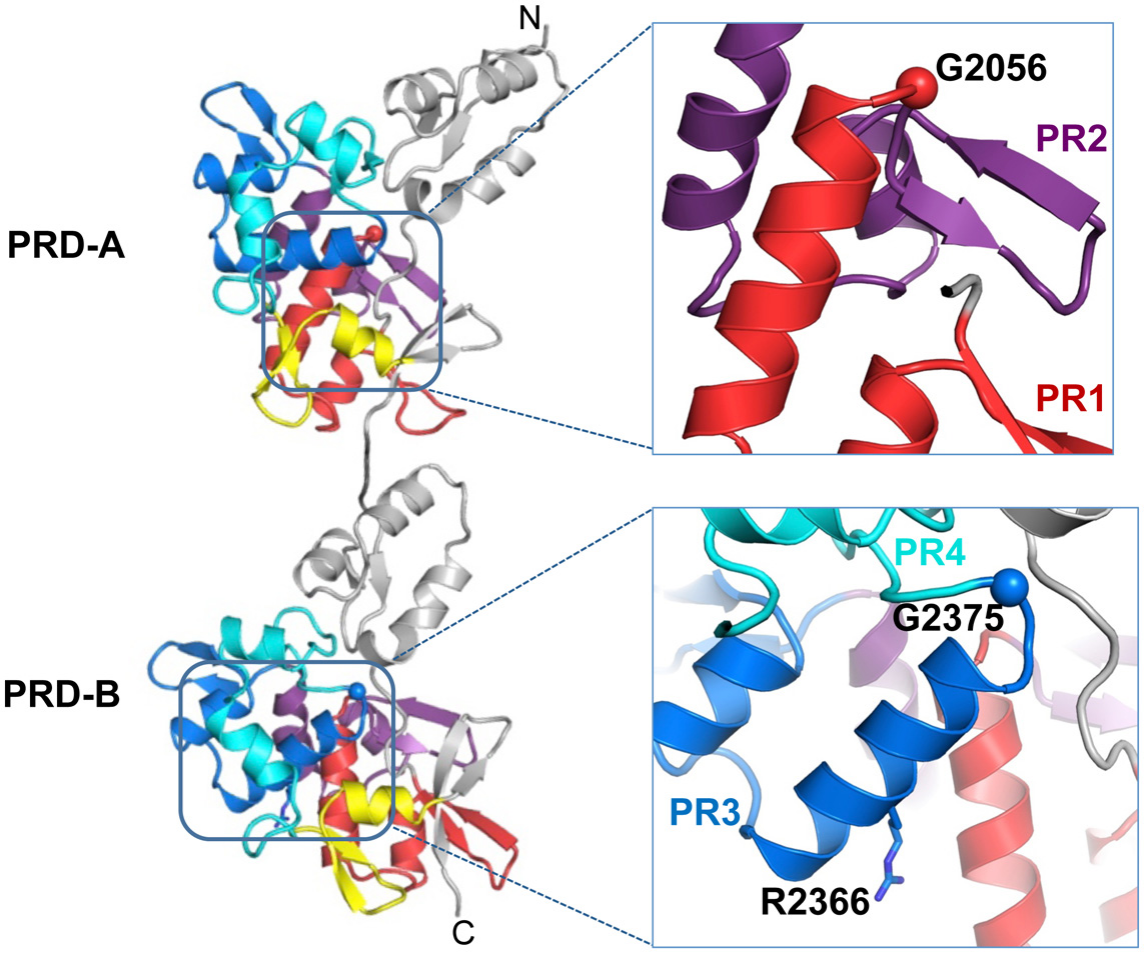
Disease-associated mutations of PRD-AB. Each plakin repeat is colored differently using the same color code as in Fig 2. Cα position of Gly residue is represented as a sphere. Two close-up views are shown on the right.

Mutations in DPCT give rise to human diseases affecting heart, skin, and hair [33, 34]. Carvajal syndrome and acantholytic epidermolysis bullosa are caused by one base deletion or nonsense mutations of desmoplakin, leading to a truncated desmoplakin lacking the PRD-C or all three PRDs [35, 36]. Skin fragility syndrome occurs in compound heterozygotes, with nonsense mutation and one missense mutation in PRD-B, R2366C. Arg2366 is located in the second α helix of PR3, and forms part of a positively charged groove with Arg2309 and Arg2385 (Fig 7). Three mutations, G2056R, G2375R and R2639Q, are associated with arrhythmogenic right ventricular cardiomyopathy (ARVC) [37]. Gly2056 and Gly2375 are located at the end of PR1 of PRD-A and at the end of PR3 of PRD-B, respectively, where the polypeptide makes a sharp turn after each of these PRs (Fig 7). Both are highly conserved in all plakin family members and appear to be important for the structural integrity of the PRD. The incorporation of Arg at this position cannot be tolerated in the structure. Arg2639 is located at the first α helix of PR1 in PRD-C and positively charged residues are favored at this position in most PRs of plakin family members. Arg2639 makes salt bridges with Asp2624 and Glu2629, which are positioned at β strands 1 and 2 of β-hairpin of PR1, and loss of positive charge by mutation to Gln at 2639 might destabilize the PR fold.

## Conclusions

Our results reveal the organization of the three IF-binding PRDs in DPCT as well as provide a high-resolution view of PRD-A. Most plakin proteins contain multiple PRDs; for example, plectin has five PRD-Bs and one PRD-C. Our analysis of the linker domain that connects PRDs B and C suggests that its N-terminal portion forms an ordered structure like that of the homologous region of periplakin and contributes to IF binding [10–12, 14]. The overall structure found here presumably enables the strong association of the multiple IF binding sites with various IFs [10, 11, 13].

The sequence following the putative structured linker region (2564–2615) has 21 Ser/Thr residues, but is not conserved in the plakin family. At least 9 to 13 Ser/Thr residues in this flexible region are predicted to be phosphorylated (NetPhos 2.0 server)(Fig 3)[38]. However, the biological importance of the length of this flexible linker and potential phosphorylation of Ser rich sequences has not been studied. Further biochemical and biophysical studies of the direct interaction between DPCT and various IF subtypes and posttranslational modifications, such as phosphorylation are required to understand the molecular mechanism of IF binding.

## Supporting Information

S1 Fig Space-filling model of PRD-AB with removal of a 4 amino acid linker. Amino acids 2204–2207 were removed from PRD-AB structure and the rest of structure was shown as space-filling model. There is no direct contact between PRD-A and PRD-B.
(TIF)

S2 Fig SAXS data analyses of PRD-AB and PRD-ABC. Raw data at four different concentrations and Kratky plots of PRD-AB and PRD-ABC are shown.
(TIF)

S3 Fig CORAL model of PRD-AB aligned to the PRD-AB crystal structure. The PRD-Bs of the two models (green) were used for the superposition. PRD-As are shown in cyan (CORAL model) and orange (crystal structure).
(TIF)
