# Supplementary figures and images for: Structure of the Intermediate Filament-Binding Region of Desmoplakin
Source: PLoS One. 2016 Jan 25;11(1):e0147641. doi: 10.1371/journal.pone.0147641 (PMC4726743; doi:10.1371/journal.pone.0147641)

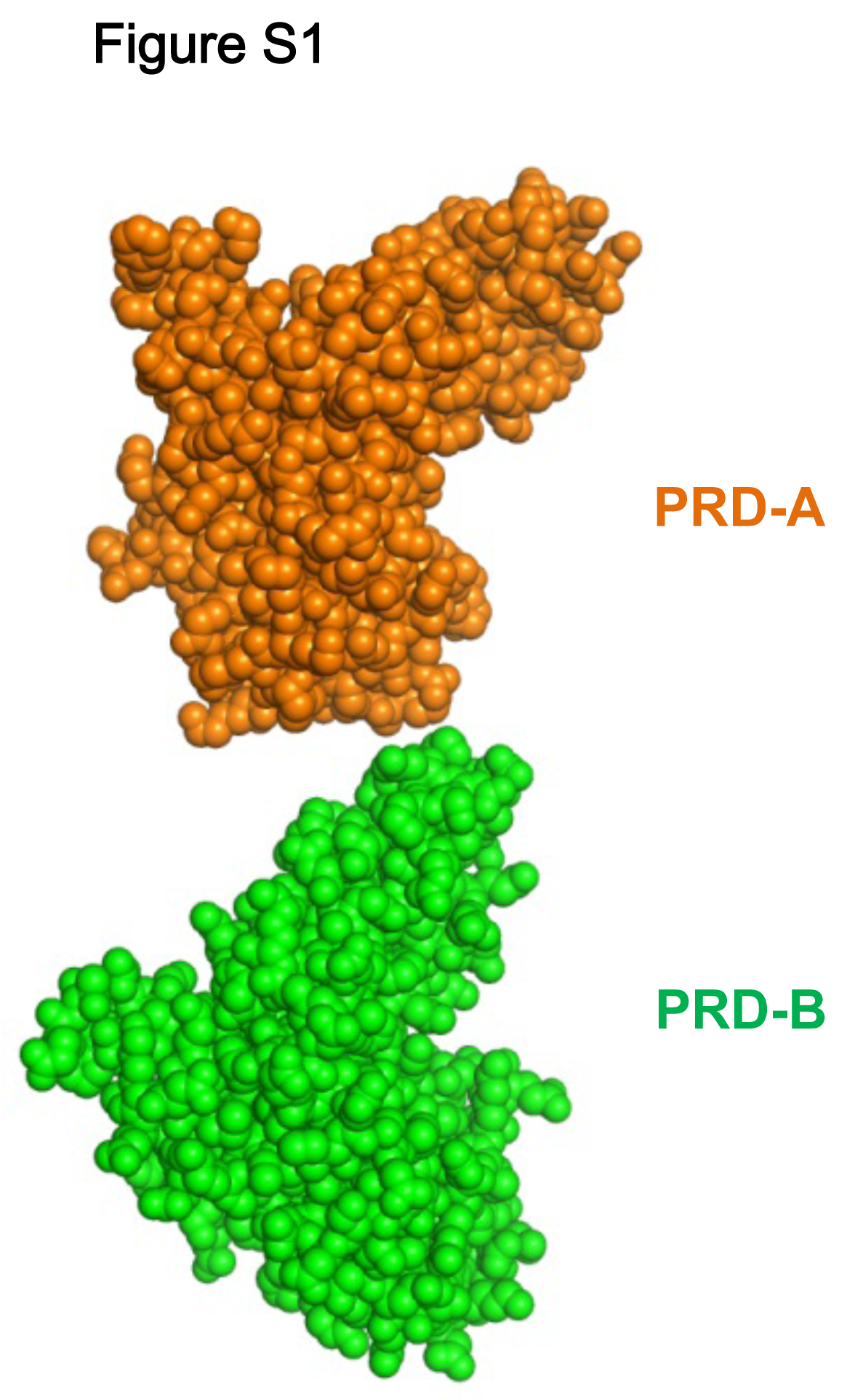

Supplement: S1 Fig — Amino acids 2204–2207 were removed from PRD-AB structure and the rest of structure was shown as space-filling model. There is no direct contact between PRD-A and PRD-B. (TIF) [file pone.0147641.s001.tif]

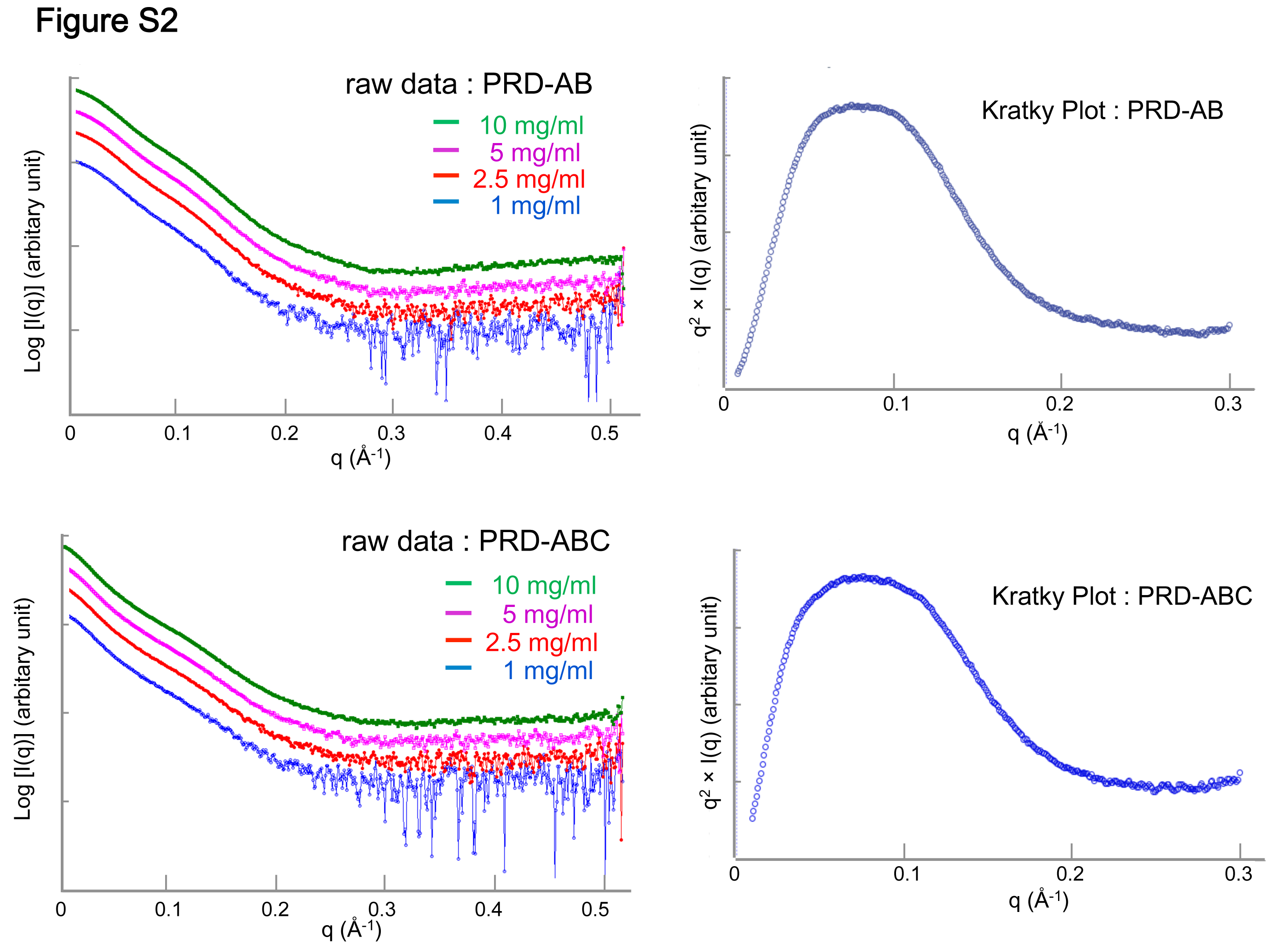

Supplement: S2 Fig — Raw data at four different concentrations and Kratky plots of PRD-AB and PRD-ABC are shown. (TIF) [file pone.0147641.s002.tif]

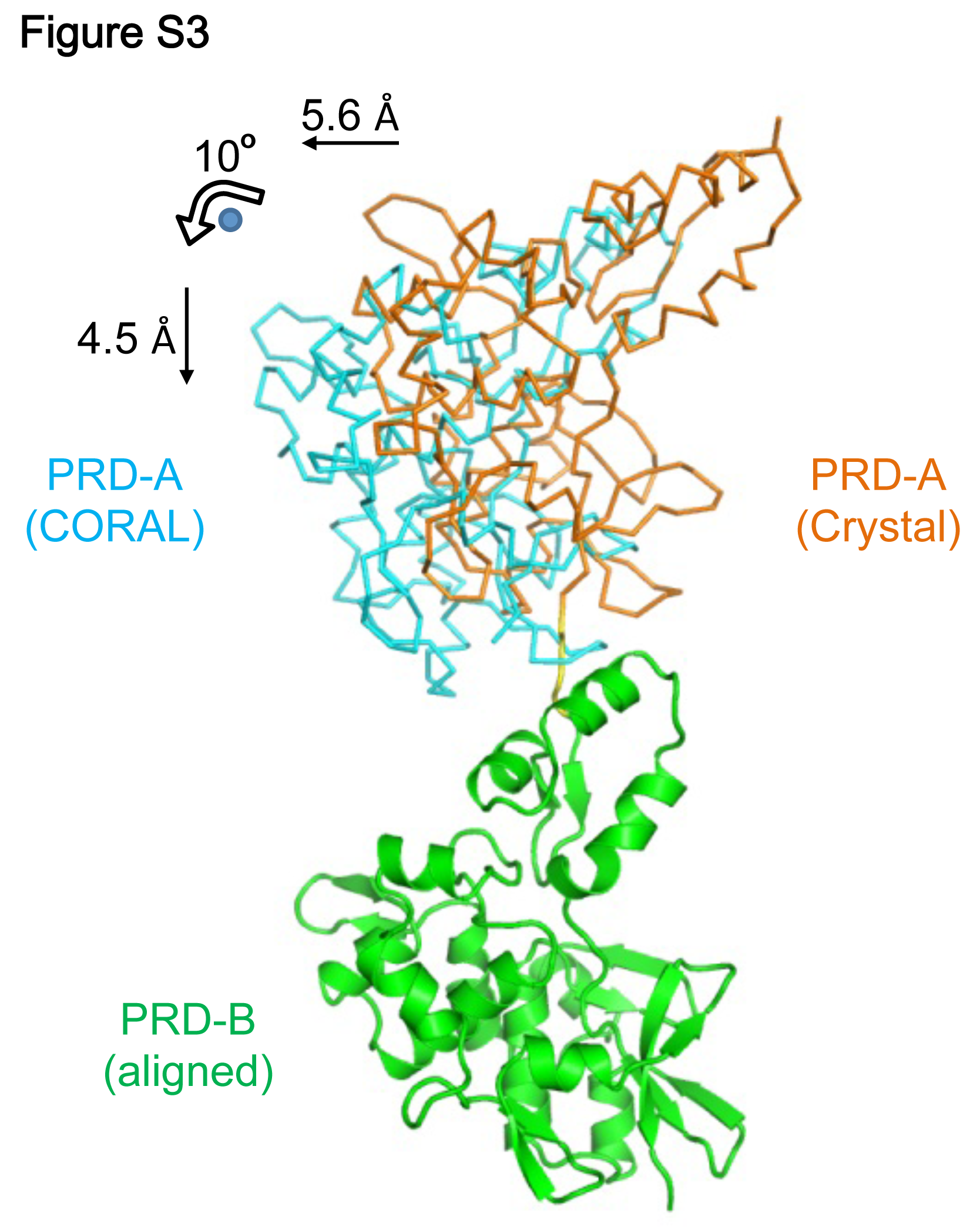

Supplement: S3 Fig — The PRD-Bs of the two models (green) were used for the superposition. PRD-As are shown in cyan (CORAL model) and orange (crystal structure). (TIF) [file pone.0147641.s003.tif]
